# What Evidence Exists to Support Palliative Allied Health Practice in Aged Care: A Scoping Review

**DOI:** 10.3390/healthcare12191973

**Published:** 2024-10-03

**Authors:** Olivia Farrer, Jennifer Tieman

**Affiliations:** End of Life Directions in Aged Care (ELDAC), Research Centre for Palliative Care, Death and Dying, College of Nursing and Health Sciences, Flinders University, Bedford Park, SA 5042, Australia; jennifer.tieman@flinders.edu.au

**Keywords:** allied health, aged care, older adult, palliative, end of life, best practice

## Abstract

**Background:** As our population ages, the demand for aged care services and palliative care is expected to increase. Allied health professionals have a diverse set of skills to offer in the management of older adults. This scoping review aimed to identify what evidence exists to support the best practice of allied health clinicians in palliative and aged care. **Methods:** Searches were conducted using broad keywords and MeSH headings with relevance to palliative, ageing and allied health care in the databases Ovid MEDLINE (R), CINAHL, EMCARE, INFORMIT, REHABDATA, PEDRO and SCOPUS, as well as the grey literature. **Results:** Only 15 studies met the inclusion criteria. A prominent finding was that regular exercise interventions delivered improved mobility, balance, sleep and quality of life outcomes when measured (*n* = 5). Broader allied health input and outcomes, such as nutrition, were not well described, other than to suggest an interprofessional approach contributed to health benefits where these were observed. **Conclusions:** The lack of research creates uncertainty about what excellent care looks like and how it can be measured, making it harder for allied health professionals to advocate for funded time in providing care at the end of life and leading to poorer outcomes for older adults.

## 1. Introduction

In recent times, there has been a shift from a clinical care model of palliative care where symptom management was the primary focus to a patient-centred approach aimed at optimising functional status and quality of life [1]. The World Health Organisation [2] has described modern palliative care as the relief of ‘health-related suffering’ that could stem from physical, psychosocial or spiritual symptoms in people who are dying. This approach should be introduced as early as possible and address palliative care needs across a range of settings, across the life course and in a multidisciplinary setting [2].

An estimated 56.8 million people worldwide need palliative care (estimate correct as of 2020), of which 25.7 million are in their last year of life [2]. Leading causes for palliative care worldwide include malignancy (28.2%), cerebrovascular disease (14.1%) and dementia (12.2%), which is similar to local Australian data where cerebrovascular and ischemic heart disease, lung cancer and dementia are the leading causes of death [3]. By 2060, the need for palliative care at the end of life is expected to double, largely attributed to adults aged over 50 years (67.1%) and older adults with multimorbidity. Multimorbidity is the occurrence of two long-term conditions such as noncommunicable diseases, which already account for almost 69% of the need for adult palliative care [2]. As our population ages, demand for both aged care services and for palliative care is anticipated to increase, and older people with multimorbidity will become a significant focus for care provision [4]. Multimorbidity is associated with lower quality of life, increased use of health services and acute care admissions and will become a significant focus for health care provision [4]. Individuals will experience progressive functional loss and increased frailty and dependency across the disease trajectory, particularly for activities of daily living [4]. Maintaining the highest level of functional ability will be an important component of palliative care for older adults moving forward [5].

The coordination of palliative care can be undertaken by a wide range of health professionals. Allied health describes a group of health professions [6] who have expertise in working within an interprofessional team and offer diverse skills related to treatment, assessment, diagnosis and counselling [7]. This article will focus on the disciplines most frequently cited as part of the multidisciplinary palliative care team in the literature, including dietitians, occupational therapists, speech Pathologists, psychologists, physiotherapists, social workers and more recently pharmacists.

Collectively, allied health makes up more than a quarter of registered health professionals in Australia; however, less than 7% are believed to be working in residential aged care [7]. Australian data show that approximately 80% of people aged 65 years and older had used aged care in the 8 years before death, and three-fifths were current clients of aged care programs (such as Home Care Packages) when they died [8]. Aged care services should consider how they can support palliative care needs and care at the end of life from within their own workforce, with the support of visiting professionals and through referral to specialist palliative care.

Given that all health professionals are likely to be involved with palliative care, allied health professionals need to be confident in delivering palliative care, and understand what they can contribute to this space, to create a better dying experience for older adults as they approach the end of life. The National Palliative Care standards (2018) have identified that workforce capability will be an important system to address in the provision of palliative care services [9] and that there is an urgent need for more rigorous research on how to best support all health professionals working in this space.

The aim of this scoping review is to present and summarise what evidence exists to support allied health best practice in providing palliative care to older adults in residential aged care or home-based aged care settings. The results of this scoping review may provide context for future research.

## 2. Methods

### 2.1. Design

The decision to use a scoping review methodology was made with the intent to summarise findings from a body of knowledge that we believed to be heterogeneous in methods and would be varied in discipline. The methodology was modelled on the Joanna Briggs Institute (JBI) manual for scoping review methodology [10]. The authors conducted a targeted database search, an online grey literature search and hand searching of reference lists. The database search was conducted in the Ovid Medline (R), CINAHL, EMCARE, Informit, REHABDATA, PEDro and Scopus databases, from 2012 to 2022, with the search taking place in August 2022. The results were restricted to the English language only. Search strings using keywords and MESH terms were developed and used for database searching (Table 1). The search strategy was then adapted for each included database, with a more simplistic keyword search in Google Scholar and Google search engine. The first 10 pages of search results (100 results) were screened for relevance, with *n* = 46 relevant documents being retrieved for full review. Additionally, professional associations for aged care and allied health peak bodies in Australia and New Zealand were searched for commentary articles relevant to the inclusion criteria that may not have been published.

### 2.2. Eligibility Criteria

The inclusion criteria were determined using the research question “What evidence exists to support palliative allied health practice, within community and residential ‘aged care’ settings?”. The criteria for inclusion were that the document described any allied health intervention for palliative care [2], either in the home (community) or a residential aged care setting, and older adults were the predominant participants being studied, i.e., participants aged >65 yrs or a mean age of participants >65 yrs. Exclusion criteria were non-allied-health interventions (e.g., nursing/physician) in an acute care, hospice or rehabilitation setting with adults <65 yrs (including mean age < 65 yrs).

### 2.3. Data Selection

**Figure 1:**
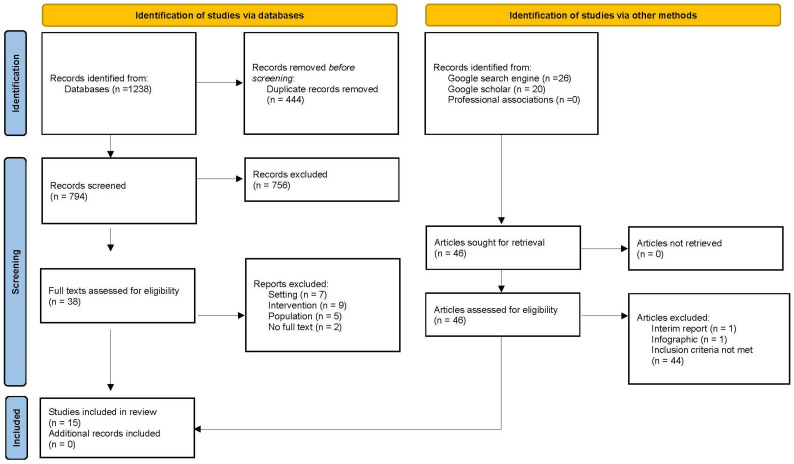
Search results in a PRISMA (Preferred Reporting Items for Systematic Reviews and Meta-Analyses) extension for scoping reviews flow diagram.

Titles and abstracts of each reference retrieved were reviewed, and final eligibility was determined by screening of full texts based on the inclusion criteria. Initial screening of titles and abstracts was conducted by the author and a member of the research team, with the second author reviewing query articles and completing the full-text screening and extraction. As grey literature articles do not typically have an abstract, articles proceeded directly into full-text screening. The scoping review yielded 1238 results, with 46 additional articles located via the grey literature search (Figure 1). Of the 84 articles selected for full-text screening, 65 did not meet the criteria largely due to having an acute care setting or population < 65 years old, or the article did not explore an allied health intervention. Finally, two studies were conference abstracts only with no full text.

### 2.4. Data Extraction

The relevant data from included documents were entered into a purpose-designed Excel spreadsheet and described according to citation, participant and allied health group, study focus and design, and key outcomes. Data extraction was completed by one reviewer (OF) and appraised using the Mixed Methods Appraisal Tool [11] (MMAT), selected to suit the varied study designs of the included papers. No quality appraisal was required for the grey literature, with no articles appropriate for inclusion. No studies were excluded based on their quality rating, due to the paucity of the literature available for review.

## 3. Results

Articles included for the review (*n* = 15) are outlined with descriptive characteristics in Table 2.

Of the articles retrieved, nine studies focused on only one discipline [12,13,14,16,19,20,21,24,26], with six including two or more allied health professions [15,17,18,22,23,25]. Physiotherapy interventions were the most frequently cited [12,14,15,18,19,20,21,23,26] (*n* = 9), followed by social work [13,17,22,23,25] (*n* = 5), psychology [17,24,25] (*n* = 3) and then nutrition and dietetics [22,25] (*n* = 2) and occupational therapy [15,16] (*n* = 2). Of the 15 studies, most originated from America (*n* = 7) and Europe (*n* = 6), with two studies from Asia. None of the included articles were research conducted in Australasia.

The studies were broadly categorised as either direct trials of allied health interventions (*n* = 8) [12,13,14,16,19,24,25,26] or descriptive outcome studies (*n* = 6) [15,17,18,20,21,23], and a qualitative study [22] (*n* = 1). The direct trial studies were predominantly a mix of randomised controlled trials [12,16,19,24,25] (*n* = 5) and pre- and post-test studies (*n* = 3) [13,14,26]. The descriptive outcome articles were all retrospective audit or cohort studies [15,17,18,20,21,23]. The results data were mainly collected over a 1–3-year period (*n* = 6) [15,18,21,23,25,26], while the remainder ranged from 1 to 6 months in duration, with no alignment of timing to study design. The studies met between two and five criteria (five being highest) for either RCT, non-randomised trials or descriptive study question sets in the MMAT appraisal score, with no study design performing better than another.

### 3.1. Direct Trials

Of the direct trials (*n* = 8) [12,13,14,16,19,24,25,26], four were physiotherapy interventions [12,14,19,26], which were typically examining outcomes for activities of daily living, balance or other physical measures, and cognitive function, with the introduction of home-based activity programs. Most of the studies were designed to be self-directed exercise programs, with a walking component. Only Cwirlej et al. (2020) [14] included additional bi-weekly home visits. Across the four studies, there were improved patient outcomes for measures such as balance and mobility [12,14,19,26], sleep [19], quality of life [14,19,23,26] and independence with self-care activities [14,19,26], with their intervention length varying from 6 [14] to 8 weeks [19] and 6 months [12] to 12 months [26].

Of the remaining four direct trials [13,16,24,25], two were studies that examined care in home packages which aimed to improve activities of daily living [16] from a single discipline (OT) or multidisciplinary input (social work, dietetics and psychologist) [25]. One study examined the benefit of advanced care planning education as part of palliative care [13] (social work), and a final study explored a novel type of psychotherapy for end-of-life preparedness [24] (psychologist).

### 3.2. Descriptive Outcome Studies

The descriptive outcome studies were retrospective audit or cohort studies [15,17,18,20,21,23] (*n* = 6). All six studies were evaluating home-based therapy or care strategies; five of the six studies were physiotherapy interventions [15,18,20,21,23], two of which made specific mention of another health profession (social work or OT) [15,23]. The sixth study was a psychologist and social work intervention to improve the psychosocial impact of the end of life for caregivers [17]. Two studies explored the impact of delivering health care at home on readmission to acute care rates [18,21].

The final study by Durepos et al. (2018) [22] qualitatively explored the benefit of structured family case conference paperwork on palliative care interventions, specifically mentioning referrals to dietetics and social workers as part of the health care approach.

An inductive approach was used to thematically organise and summarise the results from the included papers to answer the research question. The extracted results from each paper were read several times to identify similarities that could be organised into a themed grouping. Table 3 shows the studies grouped within each theme.

### 3.3. Allied Health Care in the Home vs. Usual Care

Thirteen of the studies [12,14,15,16,17,18,19,20,21,23,24,25,26] included in the scoping review explored health care in the home initiatives, with only two referencing interventions in a residential aged care setting [13,22]. Five studies [15,16,21,24,25] explored whether the location influenced health outcomes compared to usual care (defined as service delivery in an acute care or outpatient setting). Of these, five studies noted that the intervention had not resulted in a significant difference in outcomes when compared to usual care in acute or sub-acute settings [16,21,24,25]. However, a study by Cobbe et al. (2013) found that despite the activity-based program they prescribed being better tolerated by individuals in the home, there was a lower referral rate into the home-based program than for the standard outpatient service [20]. Ishani et al. (2016) [25] found their telehealth approach a feasible option for the cohort, and while this did not generate better health outcomes for their participants, it was on par with usual care delivery, which could be perceived as a positive outcome for home-based care. Similarly, discussion of results for Pilegaard et al. (2018) found that while there were no significant results for the cancer care at home intervention, participants wanted interventions that supported their activities of daily living at home [16]. In addition, Jutkowitz et al. (2012) concluded that their ‘ABLE’ program incorporating at-home and telehealth intervention from occupational health and physiotherapy clinicians was more cost effective over 6 months than the usual care approach [15].

### 3.4. Improved Daily Functioning Outcomes with Allied Health Input

Of the five studies [12,14,18,19,26] that specifically mentioned outcomes for activities of daily living, quality of life or health outcome measures, all of them noted positive outcomes. All five of the studies tested movement-based programs that could be delivered by physiotherapists either as supervised visits in the home where exercises were more tailored to the individual [14,19,26] or unsupervised in the form of a self-directed walking program [12]. Key outcomes with the introduction of an exercise program were described as improved mobility (*n* = 3) [12,14,19], balance and gait [26], cognition [12], sleep quality [19] and ability to manage activities of daily living and subsequently their quality of life [14]. Rosenburg et al. (2012) did not reference specific outcomes for activities of daily living but did note that the physiotherapy program at home had created better continuity of care and reduced frailty-related hospital readmissions by approximately 40% [18].

## 4. Discussion

The National Palliative Care strategy (2018) describes the improvement in palliative care service delivery across states and territories since the 2010 strategy was endorsed; however, there is still significant growth and improvement to be addressed [27]. In particular, the strategy is concerned with investment in the workforce, knowledge and practice of palliative care to be embedded in all care settings and ensuring that this care is individualised to the needs and preferences of the individual [27].

The number of older adults needing palliative and end-of-life care in aged care is increasing. In the last 20 years, the ageing population has increased from 12.4% to 16.3%, and this is projected to increase more rapidly over the next decade [28]. In addition, up to two-thirds of this population are likely to have three or more chronic diseases, which will give rise to higher health needs [4] and higher demands on workforce, in particular for palliative care in non-acute settings [29]. Allied health professionals are well established in the provision of care across the lifespan and make up the second-largest health workforce in Australia [29]. Local and international studies [30,31] have found that where there are sufficient palliative care services in aged care, there is reduced spending on acute care medical treatment, reduced pressure on the health care system and a better dying experience for the individual and their support network. Only one study in the scoping review measured the financial impact of delivering a team-based palliative care approach within the home [15], but it did indicate that the model was more cost-effective than inpatient or hospice usual care pathways.

Despite the clear need for allied health workforce in aged care and palliative care, currently, the engagement of AHPs in aged care is highly influenced by funding arrangements and workforce availability. Workforce data have suggested that only 5% of permanent or agency allied health were working in residential aged care or community home care support programs, and workforce availability is further reduced in rural and remote areas [7]. This will not be sufficient to meet the growing need for health care in ageing services. In this scoping review, only six of the studies explored a team approach to AHP palliative services, predominantly with exercise as the central intervention. While these studies presented positive outcomes for mobility, balance, sleep and ability to perform activities of daily living contributing to improved quality of life scores, little reference was made to the professions outside of exercise. These findings provide little opportunity to raise the profile of a broad range of allied health professions in the aged and palliative care landscape and illustrates that nuanced palliative care outcomes are important in palliative care research.

Exacerbating this issue, is an uncertainty in aged care funding models and standards regarding the roles and responsibilities of AHPs delivering palliative care, including end-of-life care [29] and how the team care approach is facilitated through these models. A lack of visibility and acknowledgement of specific AHP roles in the literature is likely to impact on whether professions are acknowledged in funding instruments and how we train and upskill AHPs from the outset. There is risk that this becomes a cyclical problem, where there is limited research on which to base best practice as an AHP, and low visibility in the added value that AHPs can bring to palliative and end-of-life care.

The studies discussed in this scoping review, while limited, do suggest that older adults see improvements in quality of life and measures of activities of daily living from a reablement approach to palliative care broadly related to AHP input. Service provision in the home, whether community-based or residential aged care, seems preferential to hospice or acute care settings. This is consistent with the broader literature, which notes that health professional presence, in the form of availability and home visits, and their competence in the sense of effective symptom control and skilful communication have been identified as important for both patients and family caregivers to feel a sense of security [32].

Overall, this scoping review has identified a paucity of the literature around successful health interventions for all allied health disciplines, making it difficult to discuss their broad scope of practice in aged and palliative care specifically. [9]. Without evidence specific to palliative care and interprofessional practice for an ageing cohort, clinicians are relying on generalised information to support practice. The implications of this are that practice may not be sufficiently attuned to the modern palliative care approach or the needs of older adults with multimorbidity in an aged care context. An investment in training and research to inform best practice, increase practitioner confidence to deliver palliative services and grow the workforce is needed. Subsequently, a framework for implementing any new best practice and associated evidence would need to be considered.

As with all scoping studies, there are limitations to the usefulness of these findings. The methodology does not address the issue of ‘synthesis’; rather, this study provides a descriptive and thematic account of available research. However, the search was designed and executed by an expert librarian, and the scoping methodology was adopted from an established framework [10]. Studies were restricted to the English language only, but by not restricting included studies based on quality appraisal, the study included a greater range of study designs and methodologies than a systematic review, which in view of the limited literature, strengthens the rationale for a scoping approach. The lack of research in palliative and aged care is seen more broadly than in just the allied health literature, and this will have an impact on the evidence base to support clinical decision making and policy [33]. These findings would suggest a need for investment in a research framework that seeks to better understand the contributions of AHPs in palliative care in the aged care context.

## 5. Conclusions

The aged care landscape is changing; more older adults will want to receive palliative services in their own homes and residential aged care. Allied health professionals are actively working in this area, but the workforce is insufficient to meet the growing demand for ageing and palliative care services. The lack of research creates uncertainty in what excellent care looks like and how it can be measured and will make it harder for AHPs to advocate for funded time within the palliative care team. The risk for not addressing the shortfalls in allied health palliative care delivery will be poorer outcomes for older adults, including highly variable practice and limited quality measures for workforce skill development, and economic burden as frequent use of acute care services will continue. It will be important to upskill the workforce to support older adults in their end of life, and it is evident that more research is needed to achieve this outcome.

## Figures and Tables

**Table 1:** List of keyword search and string terms adapted for each database.

(exp Allied Health Personnel OR allied health OR Dietetics OR Dietitian OR Physiology and Exercise OR Occupational Therapy OR Physical Therapy Modalities OR Psychology OR Speech language Pathology AND Death OR Dying OR End of Life OR EOL OR End stage care OR Palliative Care or Terminal Care or Terminally Ill or Advance Care Planning or Advance Directive OR Suicide OR Supportive Care or Bereavement or Grief or Hospice AND Home care services OR Health services for the aged OR Residential care OR Aged care or Community based Care or Senior Living or Senior Centre or Retirement home OR Long term care OR Transitional Care or Care home or Assisted Living OR Skilled nursing facility OR Home nursing or In home care or In home support AND Aged OR Aged 80 and over OR Geriatrics OR Senior OR Elder or elders or Elderly OR Centenarian or (Aged 50 or aged 55 or aged 60 or aged 65 or aged 70 or aged 75 or aged 80 or aged 85). Limit to (English language and yr.= “2012–Current”)

**Table 2:** Overview of allied health interventions in the palliative management of older adults in a community or residential aged care (RAC) setting from 2012 to 2022.

Author, Year	Sample (*n*)	Setting	Allied Health	Study Aim	Study Design/Timing	Allied Health Intervention	Key Outcomes
Baggetta et al. [12], 2018 Italy	53	Home	Physiotherapy	Efficacy and safety of a home-based (unsupervised) activity program for ESRD patients	Secondary analysis of RCT, 6 months	Walking exercise plan provided. A 6 min walking distance and sit-to-stand test at baseline and 6 months.	A physical activity program improves physical performance and stabilises cognitive function in dialysis patients > 65 yrs, when compared to a control group.
Chan et al. [13], 2021 Hong Kong	128	RAC	Social work	To examine the impact of an advance care planning group education program delivered weekly in 1.5 h sessions	Quasi-experimental, 6 weeks	A 1.5 h education session delivered weekly on physical, psychological and social care.	No statistically significant change in QOL measures after groups, nor in EOL preferences. However, awareness of advanced care increased, and there was a slight increase in advance directive completions and family discussions.
Ćwirlej-Sozańska et al. [14], 2020 Poland	60	Home	Physiotherapy	To assess the impact of a multi-component physiotherapy program delivered twice weekly over 6 weeks	Pre- and Post-Test 6 weeks	Twice-weekly input (45 min sessions) working towards individualised goals.	A statistically significant improvement was observed in the patients in many areas including ADLs, mobility, balance and reduction in depression and an increase in QOL. Particularly important was the improvement in independence with self-care activities.
Jutkowitz et al. [15], 2012 USA	319	Home	Occupational therapy (OT) and physiotherapy	Evaluating the cost-effectiveness of ageing in places using the ‘ABLE’ program	Retrospective cohort study 12 months	First 6 months, 4× in-person OT visits (1.5 h) and 1 telehealth + 1 × 90 min physiotherapy visit. Maintenance 6 months, 3× OT telehealth.	ABLE provides semi-intensive OT and physiotherapy at home over 6 months, with telehealth support in the subsequent 6 months. The total cost of ABLE per person is AUD 942 vs. usual care at AUD 13,179 when modelled using mortality data.
Pilegaard et al. [16], 2018 Denmark	121	Home	Occupational therapy (OT)	Evaluating a ‘Cancer Home Life Intervention’ (ADL performance as the key outcome)	RCT, 3 months	Between 1 and 3 home visits (over 1–3 weeks, <120 min each) + 1–3 telehealth follow-up contacts.	An occupational-therapy-based adaptive program for people with advanced cancer. The program was tailored to individuals incorporating F2F and telehealth. No significant results were observed compared to usual care.
Pompili et al. [17], 2014 Italy	122	Home	Psychologist and social work	Evaluating a home assistance program in patients with glioblastoma multiforme	Retrospective cohort study	Program included weekly team meetings with a dedicated team manager and home visits as required.	A positive psychosocial impact on caregivers from home visits by the psychologist and social worker was observed, particularly education around mobilising, texture mod nutrition, exercises and respiratory care. QOL scores also improved.
Rosenburg et al. [18], 2012 Canada	248	Home	Physiotherapy (component of broader health team)	To evaluate the ‘primary integrated elder care at home (PIECH)’ program for success in reducing hospital admissions in frail older adults	Retrospective cohort study, 12 months	Physiotherapy home visits ranged from daily for short periods to 1–3 times over 4–6 weeks.	The program created better continuity of care for the patient. Patients saw the physiotherapist at least once. A significant reduction in hospital admissions was observed.
Cheville et al. [19], 2013 USA	56	Home	Physiotherapy	Evaluation of the ‘REST’ program in adults with Stage IV cancer	RCT, 8 weeks	Initial in-person appointment (90 min), with a biweekly telehealth review.	A short, focused home exercise program in association with a progressive pedometer-based walking program appears capable of improving the mobility, sleep quality and fatigue of patients with Stage IV lung and colorectal cancer.
Cobbe et al. [20], 2013 Ireland	103	Home	Physiotherapy	Review of referral process for at-home palliative care physiotherapy	Retrospective audit, 6 months	In-home physiotherapy appointments, most commonly for education (71%), gait re-education (43%) and exercise (72%).	Almost a quarter of all palliative care referrals came to physiotherapy, which is a lower rate compared to in-hospital physiotherapy. There was a seemingly poor understanding from nurses of the role of physiotherapy in palliative care. Despite this, home-based patients generally tolerated a broader range of activity input when referred.
Cousse et al. [21], 2019 France	62	Home	Physiotherapy	Evaluating the readmission rate of patients with COPD following a home discharge program including physiotherapy	Retrospective cohort study, 2015–2017 (2 yrs)	Physiotherapist home visit within 1 week of discharge from acute care (no further intervention details).	The home discharge program did not lead to an improvement in readmission or death rate compared to standard care, but patients in the intervention had more severe COPD to start with, which may skew outcomes. No QOL outcomes were recorded.
Durepos et al. [22], 2018 Canada	39	RAC	Dietetics and social work	To evaluate the structure and multidisciplinary involvement in family care conferences	Qualitative/content analysis	Monthly project team meetings to upskill on project delivery and the use of standardised forms.	Palliative care teams trained to participate in multidisciplinary family conferences using standardised documentation helped facilitate timely interventions around EOL care, physical issues and social care.
Fleischer et al. [23], 2018 USA	85	Home	Social work and movement disorder fellow	Exploration of outcomes for home-based interprofessional interventions for older adults with Parkinson’s disease, “The Edmond J Safra Interdisciplinary Home Visit Program for Advanced Parkinsons Disease”	Retrospective cohort study, 2014–2016	First home visit conducted by a social worker to create a plan and take baseline measures. Follow-up via telehealth after 2 weeks, then every 4 months. Other allied health referred as required. The health team meet weekly to discuss patients enrolled in the program.	A median of three visits to patients were recorded, and additional therapy referrals (particularly speech pathology and dietetics) were made at most visits. High satisfaction with home care and better medication titration/management was noted. Models demonstrated the ability to reach vulnerable clients.
Fraguell-Hernando et al. [24], 2019 USA	32	Home	Psychologist	A comparison of IMCP-PC therapy with usual psychotherapy in palliative oncology patients	Two-arm randomised feasibility trial	A total of 3 × 45–60 min therapy sessions across 1 month	The post-treatment questionnaire revealed no significant between-group differences regarding patient perception of the structure, focus or length of treatment. However, the IMCP-PC group rated the treatment more highly with regard to its value in helping them to find meaning in life.
Ishani et al. [25], 2016 USA	301	Home	Social work, psychologist and dietetics	Evaluating a customised self-management program for CKD patients at home utilising an interprofessional team care telehealth approach	RCT, 12 months	Weekly telehealth after initial in-home appointment. Allied health referred as required for home visits, and feedback was provided to the team at weekly meetings.	Telehealth by an interprofessional team is a feasible care delivery strategy in patients with CKD but is not statistically better than usual care.
Mehta et al. [26], 2020 India	44	Home	Physiotherapy	Evaluating a home-based activity program for older adults with Parkinson’s disease	Pre- and post-test, 12 months	Home-based activity program (45–60 min per visit) delivered by physiotherapy. Typical treatment length was 74 days, with a mean of 66.8 visits during this time.	Home-based physiotherapy interventions like stretching exercises, active assisted, active exercises, strengthening exercises, balance and gait training were found to have a positive effect on Berg balance scores among this group of patients.

Abbreviations: RAC = residential aged care; RCT = randomised controlled trial; EOL = end of life; QOL = quality of life; ADL = activity of daily living; IMCP-PC = Individual Meaning-Centred Psychotherapy—Palliative Care.

**Table 3:** Articles grouped by outcome theme.

Theme	Study
Allied health care in the home vs. usual care	Pilegaard et al., (2018) [16], Cousse et al. (2019) [21], Cobbe et al. (2013) [20], Fraguell-Hernando et al. (2019) [24], Jutkowitz et al. (2012) [15], Ishani et al., (2016) [25]
Improved daily functioning outcomes with allied health input	Bagetta et al. (2018) [12], Cwirlej-Sozanska et al. (2020) [14], Rosenburg et al. (2012) [18], Cheville et al. (2013) [19], Mehta et al. (2020) [26]
